# Comprehensive Transcriptomic Analysis of the Molecular Mechanisms Conferring Resistance to Rice Blast in the Elite Restorer Line Fuhui2165

**DOI:** 10.3390/ijms262010164

**Published:** 2025-10-19

**Authors:** Shuijin Zhang, Yinyin Mao, Yonghe Hong, Feiyan Zheng, Ronghua Hu, Shihang Tu, Fantao Zhang, Peng Zhou

**Affiliations:** 1 Rice Research Institute, Fujian Academy of Agricultural Sciences, Fuzhou 350002, China; 2 College of Life Sciences, Jiangxi Normal University, Nanchang 330022, China

**Keywords:** rice blast, Fuhui2165, transcriptome, differential expressed genes, disease resistance mechanism

## Abstract

Rice blast, caused by *Magnaporthe oryzae* (*M. oryzae*), severely threatens global rice production with substantial yield losses, endangering food security and driving demand for resistant varieties. Fuhui2165 (FH2165), an elite restorer line with stable blast resistance, superior agronomic traits, and high grain quality, is valuable for hybrid breeding, but its resistance mechanisms remain unclear. In this study, we investigated the rice blast resistance and underlying mechanisms in FH2165 and its parental lines (Huahangsimiao/HHSM, Minghui86/MH86, and Shuhui527/SH527) using transcriptome sequencing analysis. Phenotypic analysis revealed that FH2165 and HHSM exhibited stronger resistance compared to MH86 and SH527. Differential expression analysis identified 3886, 2513, 3390, and 4678 differentially expressed genes (DEGs) in FH2165, HHSM, MH86, and SH527, respectively. Gene Ontology (GO) enrichment analysis highlighted DEGs associated with chloroplasts, plastids, thylakoids, and related cellular components. Kyoto Encyclopedia of Genes and Genomes (KEGG) analysis identified significant enrichment in pathways such as carbon metabolism, amino acid biosynthesis, and photosynthesis. This suggested that defense strategies could involve energy reprogramming and the synthesis of secondary metabolites. Additionally, the DEGs co-expressed specifically in FH2165 and HHSM were enriched in functions related to RNA processing, GTP binding, and L-ascorbic acid binding, with purine metabolism playing a role in the regulation of energy and signaling. These findings elucidated the critical metabolic and signaling networks that underlie the blast resistance of FH2165 and offered potential targets for breeding high-yield, disease-resistant hybrid rice varieties.

## 1. Introduction

Rice serves as a fundamental food crop, supporting over half of the global population. However, its production is significantly jeopardized by rice blast, a destructive disease caused by the fungus *Magnaporthe oryzae* (*M. oryzae*) [1]. Commonly referred to as rice cancer, this disease affects all rice plant tissues throughout the growth cycle, resulting in average yield losses ranging from 10% to 30%, and in severe outbreaks, can lead to complete crop failure [2]. The widespread prevalence of rice blast, particularly in regions of Asia and Africa that are heavily reliant on rice cultivation, presents a substantial challenge to achieving stable and high-yield rice production, thereby threatening global food security [3]. Current management strategies predominantly depend on chemical fungicides, such as tricyclazole and isoprothiolane, which not only elevate production costs but also contribute to environmental pollution, leave pesticide residues in grains, and promote the development of fungicide resistance among pathogens [4,5]. Consequently, there is an urgent need for the development of sustainable solutions to control rice blast.

Since the successful cloning of the first blast resistance gene *Pib*, over 50 resistance genes have been identified and integrated into breeding programs, leading to a significant reduction in disease incidence and the stabilization of rice production on a global scale [6,7,8]. These genes can be categorized into three primary classes: (1) NBS-LRR proteins, where the LRR domain determines the resistance spectrum, and the HMA domain influences pathogen recognition specificity; (2) receptor-like kinases (RLKs), which are characterized by extracellular ligand-binding and intracellular kinase domains; and (3) ARM repeat proteins, whose number of repeats is positively correlated with the breadth of resistance. Although a few of cloned genes provide broad-spectrum resistance, most display narrow specificity, allowing pathogens to quickly evolve and circumvent these defenses, thus constraining the long-term effectiveness of disease management strategies. Consequently, it is imperative to broaden rice germplasm screening, especially among indigenous varieties and wild rice resources, and to investigate novel resistance genes and their underlying mechanisms to enhance research on blast resistance.

Fuhui2165 (FH2165), an elite restorer line developed by the Rice Research Institute of the Fujian Academy of Agricultural Sciences, China, exhibits superior agronomic characteristics, including optimal plant and leaf morphology, strong combining ability, high grain quality, and robust resistance to blast disease [9]. The breeding process of FH2165 involved a three-way cross between Huahangsimiao (HHSM) and Minghui86 (MH86), followed by backcrossing with Shuhui527 (SH527) and multi-generational selection for blast resistance up to the F_11_ generation. FH2165 has been effectively utilized in the development of many elite hybrid cultivars such as Qiyou2165, Taiyou2165, and Yingfengyou2165, all of which demonstrate excellent quality and blast resistance [9]. Nonetheless, the molecular mechanisms responsible for its agronomic traits, particularly its blast resistance, remain inadequately understood. A deeper understanding of these mechanisms is crucial for germplasm enhancement and hybrid breeding, thereby promoting the effective utilization of FH2165 in rice production.

Transcriptomics serves as a powerful tool for elucidating the mechanisms underlying rice blast resistance [10]. Through comparative genome-wide expression profiling, researchers can reveal dynamic regulatory networks involved in host–pathogen interactions, thereby expediting the discovery of resistance genes and advancing molecular breeding efforts. Transcriptome sequencing, a fundamental technique within transcriptomics, has facilitated the identification of key resistance-related genes and their functional roles in rice blast studies. For example, Iqbal et al. conducted a comparative transcriptome and genome analysis between the susceptible Zhefang rice variety Diantun502 and its resistant counterpart Diantun506 following infection by *M. oryzae* [11]. Yang et al. performed transcriptomic analyses on the elite rice variety Huizhan, providing insights into its disease resistance and heat tolerance [12]. Similarly, Zheng et al. performed transcriptomic analyses to elucidate the regulatory network of chitin-elicitor binding protein (CEBiP) in rice defense against *M. oryzae* [13]. In this study, we utilized transcriptome sequencing to analyze the blast defense responses of FH2165. The main objectives were to identify genes associated with disease resistance, elucidate their underlying mechanisms, and inform the safe application of this elite restorer line in rice cultivation.

## 2. Results

### 2.1. Rice Blast Resistance Phenotypes

**Figure 1:**
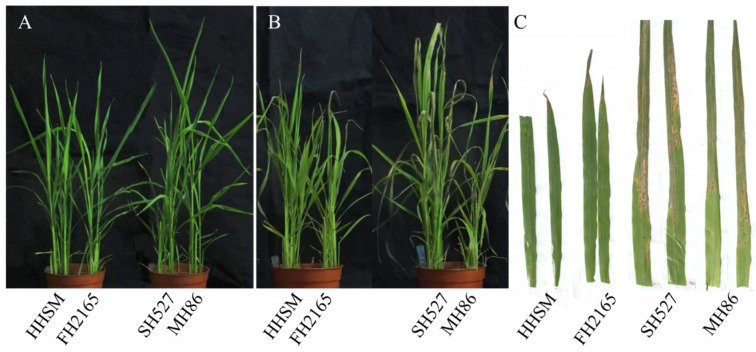
Impact of *M. oryzae* on FH2165 and its parent varieties. (**A**) the untreated control group; (**B**) the treated group; (**C**) leaf growth post-treatment.

To evaluate the resistance of rice blast, we conducted a comparative analysis of the infection phenotypes of FH2165 and its three parental varieties (HHSM, MH86, and SH527) following inoculation with *M. oryzae*. In the untreated control group, all rice plants exhibited healthy growth and normal leaf development. However, after inoculation and a subsequent period of cultivation, all varieties exhibited different levels of leaf curling. Meanwhile, FH2165 and HHSM exhibited only mild curling and a limited number of disease lesions, whereas MH86 and SH527 demonstrated severe leaf curling accompanied by prominent blast lesions (Figure 1). These phenotypic observations suggested that FH2165 and HHSM possess greater resistance to rice blast compared to the varieties MH86 and SH527.

### 2.2. RNA Sequencing and Alignment

According to previous studies, *M. oryzae* completes a series of critical infection steps within 24 hpi: conidial germination, followed by appressorium formation, penetration of the rice leaf epidermis, and initial hyphal colonization [14,15]. Consistently, 24 hpi was the time point where clear phenotypic differences between resistant and susceptible genotypes emerged in this study (Figure 1). Therefore, to elucidate the molecular characteristics underlying the rice blast resistance of FH2165 and to identify the core regulatory pathways involved in its defense response, RNA sequencing was conducted on eight plant samples: FH2165 (FHMO_0), HHSM (HHMO_0), MH86 (MHMO_0), and SH527 (SHMO_0) at 0 hpi, as well as their corresponding samples at 24 hpi (FHMO_24, HHMO_24, MHMO_24, SHMO_24). Sequencing performed on the Illumina platform yielded between 46,636,886 and 51,407,562 raw reads per sample (Table 1). Following quality control procedures, which included the removal of adapters, low-quality reads, and reads with more than 10% ambiguous bases, between 41,370,362 and 47,124,988 high-quality clean reads were retained (Table 1). Quality assessment confirmed the reliability of the sequencing data, with Q20 scores (indicating the percentage of bases with a Phred quality score of at least 20) ranging from 98.15% to 98.27%, Q30 scores (indicating the percentage of bases with a Phred quality score of at least 30) ranging from 94.51% to 94.83%, and GC content varying from 48.15% to 49.66% across all samples (Table 1). The clean reads were aligned to the rice reference genome, and the alignment achieved unique mapping rates between 91.14% and 92.99% for all samples (Table 1). These metrics demonstrated the high quality of the transcriptome data, rendering it suitable for subsequent analyses.

### 2.3. Identification of Differentially Expressed Genes (DEGs) by RNA-Seq

**Figure 2:**
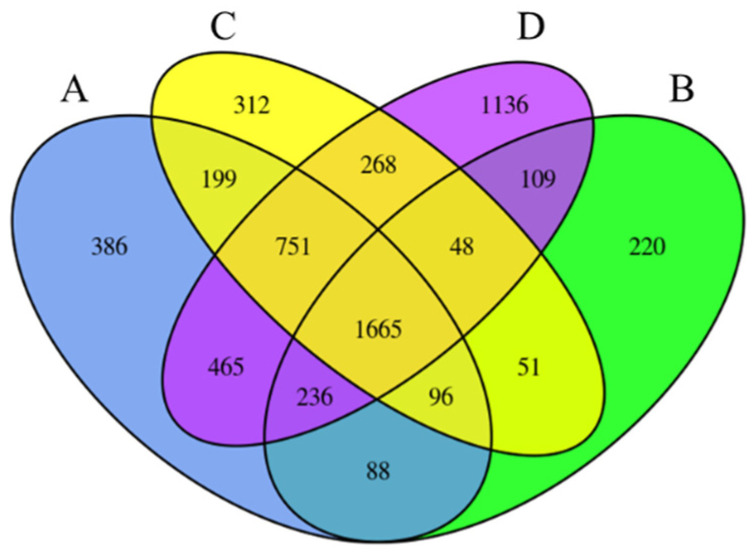
The Venn diagram illustrating the overlap of DEGs among various groups. (**A**) FH2165; (**B**) HHSM; (**C**) MH86; (**D**) SH527.

To elucidate the genetic mechanisms underlying the rice blast response, differential expression analysis was conducted utilizing DESeq. This analysis compared samples inoculated with *M. oryzae* at 24 hpi with non-inoculated control samples at 0 hpi. The analysis revealed the following differentially expressed genes (DEGs) across various rice cultivars: FH2165 exhibited 3886 DEGs, with 2009 being upregulated and 1877 downregulated (Table S2); HHSM presented 2513 DEGs, comprising 1230 upregulated and 1283 downregulated (Table S3); MH86 demonstrated 3390 DEGs, with 1805 upregulated and 1585 downregulated (Table S4); and SH527 displayed the highest number of DEGs at 4678, with 2623 upregulated and 2055 downregulated (Table S5). Notably, HHSM exhibited the fewest DEGs, suggesting distinct gene expression profiles among the four cultivars in response to *M. oryzae* infection. A Venn diagram was employed to discern shared and cultivar-specific DEGs among FH2165 and its three parental lines (Figure 2). The analysis identified 386 DEGs specific to FH2165, 220 specific to HHSM, 312 specific to MH86, and 1136 specific to SH527. In addition, a total of 1665 DEGs were consistently expressed across all four rice varieties, suggesting the presence of conserved genetic elements potentially involved in plant-pathogen interactions. The variety FH2165 shared 2085 DEGs with HHSM, 2711 with MH86, and 3117 with SH527. These extensively shared DEGs suggested overlapping mechanisms of blast response between FH2165 and its parental lines, with these co-expressed genes representing promising candidates for further investigation into genes critical for blast resistance.

To validate the reliability of the transcriptome sequencing, the expression levels of 10 randomly selected DEGs (comprising 5 up-regulated and 5 down-regulated genes) were quantified using qRT-PCR, with the rice *Actin1* gene serving as the internal reference. The qRT-PCR results were compared with the gene expression profiles obtained from RNA-Seq analysis. All transcripts exhibited consistent expression trends across both analyses, thereby confirming the robustness of the RNA-Seq results (Figure S1).

### 2.4. Functional Classification via Gene Ontology (GO) Analysis

To elucidate the gene functions in FH2165 and its three parental varieties during infection by *M. oryzae*, we conducted a Gene Ontology (GO) annotation analysis on DEGs across four selected comparison groups: FHMO_0 vs. FHMO_24, HHMO_0 vs. HHMO_24, MHMO_0 vs. MHMO_24, and SHMO_0 vs. SHMO_24. This analysis aimed to identify significantly enriched GO terms. In FH2165, a total of 3281 DEGs were significantly enriched across 450 GO terms, comprising 300 terms in biological processes (BP), 60 in cellular components (CC), and 90 in molecular functions (MF) (Table S6). The HHSM variety exhibited 2116 DEGs enriched in 392 significant GO terms (BP: 267; CC: 48; MF: 77) (Table S7). In MH86, 2858 DEGs were enriched in 487 GO terms (BP: 331; CC: 58; MF: 98) (Table S8). SH527 presented the highest number of DEGs, with 3910 enriched in 504 GO terms (BP: 324; CC: 87; MF: 93) (Table S9).

**Figure 3:**
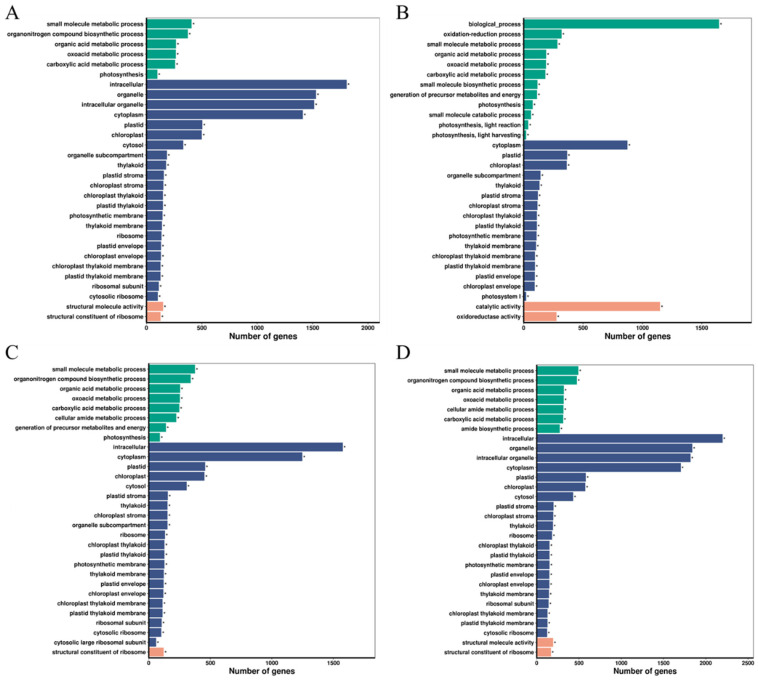
Functional classification of the top 30 enriched Gene Ontology (GO) terms in FH2165 and its parental lines. (**A**) FH2165; (**B**) HHSM; (**C**) MH86; (**D**) SH527. GO categories are color-coded: green represents biological processes, blue represents cellular components, and orange-red represents molecular functions. * denotes significantly enriched GO terms.

Specifically, in FH2165, 2538 DEGs within the BP category were primarily annotated to cellular processes, metabolic processes, biosynthetic processes, oxidation-reduction processes, and responses to stimuli. Within the CC category, 2522 DEGs were mainly associated with organelles, cytoplasm, plastids, and organelle membranes. Regarding MF, 2749 DEGs were predominantly annotated to catalytic activity, ion binding, oxidoreductase activity, and RNA binding. The GO terms were also significantly enriched in the three parental lines, indicating these GO terms’ potential involvement in fundamental metabolic processes associated with rice blast resistance. Notably, the majority of the top 30 terms in FH2165 and its parental lines were categorized under Cellular Component (CC), with many conserved CC terms (such as chloroplast, plastid, cytoplasm, thylakoid, and thylakoid membrane) enriched across all four groups (Figure 3). These findings suggested that blast resistance in FH2165 could be regulated by DEGs linked to these CC terms.

### 2.5. Kyoto Encyclopedia of Genes and Genomes (KEGG) Pathway Mapping

Genes collaborate to execute biological functions within organisms, and KEGG enrichment analysis serves to identify the principal biochemical metabolic and signal transduction pathways implicated in DEGs. To elucidate the metabolic regulatory networks of DEGs during *M. oryzae* infection across four rice groups, we aligned the identified DEGs with the KEGG database and employed hypergeometric tests to ascertain significantly enriched pathways. Consequently, DEGs in the FH2165 group were enriched across 124 metabolic pathways, followed by HHSM with 120 pathways, MH86 with 123 pathways, and SH527 with 128 pathways (Tables S10–S13). Notably, FH2165 and its three parental lines exhibited shared enrichment in several critical pathways characterized by a substantial number of DEGs, including carbon metabolism, glyoxylate and dicarboxylate metabolism, carbon fixation in photosynthetic organisms, photosynthesis-antenna proteins, biosynthesis of amino acids, pentose phosphate pathway, citrate cycle (TCA cycle), and photosynthesis. Among these pathways, secondary metabolite biosynthesis, carbon metabolism, and amino acid biosynthesis pathways harbored the greatest number of enriched DEGs.

**Figure 4:**
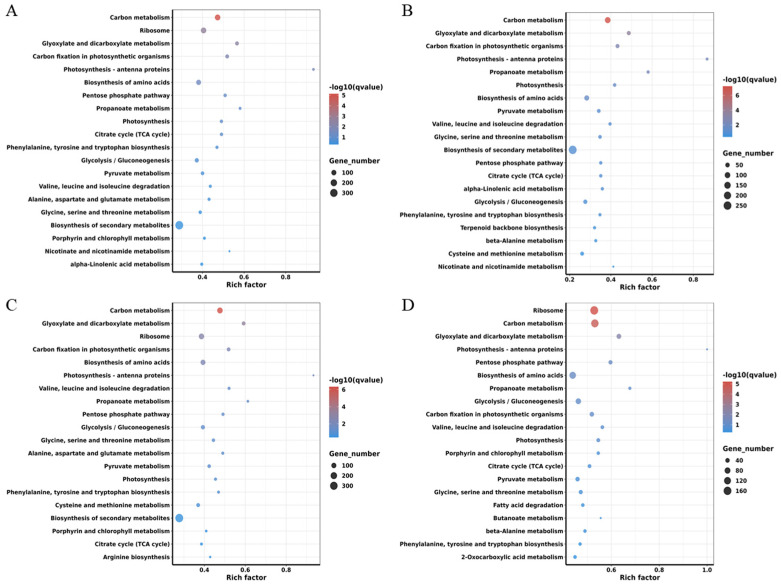
KEGG pathway enrichment analysis of the top 20 most enriched metabolic pathways for DEGs in FH2165 and its three parental lines: (**A**) FH2165; (**B**) HHSM; (**C**) MH86; (**D**) SH527.

A noteworthy observation was the differential enrichment of the ribosome pathway among the different groups. Specifically, FH2165, MH86, and SH527 demonstrated substantial enrichment of DEGs within this pathway, with 128 (105 upregulated, 23 downregulated), 122 (115 upregulated, 7 downregulated), and 167 (135 upregulated, 32 downregulated) DEGs, respectively. In contrast, HHSM exhibited only 22 DEGs (4 upregulated, 18 downregulated) in the ribosome pathway, indicating no significant enrichment. These findings suggested a minimal impact of rice blast on the ribosome pathway in HHSM, implying that blast resistance could depend on distinct pathways and regulatory mechanisms across different rice varieties. Furthermore, the analysis of the top 20 most enriched metabolic pathways for DEGs across the four groups (Figure 4) underscores both conserved and genotype-specific pathway associations.

### 2.6. Analysis of Mitogen-Activated Protein Kinase (MAPK) Signaling Pathway

The Mitogen-Activated Protein Kinase (MAPK) signaling pathway is integral to plant immune responses against pathogens [16]. Specifically, infection by *M. oryzae* triggers MAPK cascades in rice, which subsequently modulate the expression of defense-related genes and programmed cell death. Consequently, we conducted a detailed analysis of the DEGs associated with MAPK signal transduction. Within the MAPK signaling pathway-plant category, the FH2165 line exhibited 39 enriched DEGs, comprising 26 upregulated and 13 downregulated genes. In comparison, the HHSM line demonstrated 30 DEGs (23 upregulated, 7 downregulated), the MH86 line showed 31 DEGs (22 upregulated, 9 downregulated), and the SH527 line presented 40 DEGs (26 upregulated, 14 downregulated). Notably, across FH2165 and its parental lines, the number of upregulated genes within this pathway surpassed that of downregulated genes, indicating that these upregulated genes could encompass key regulators of rice blast resistance.

**Figure 5:**
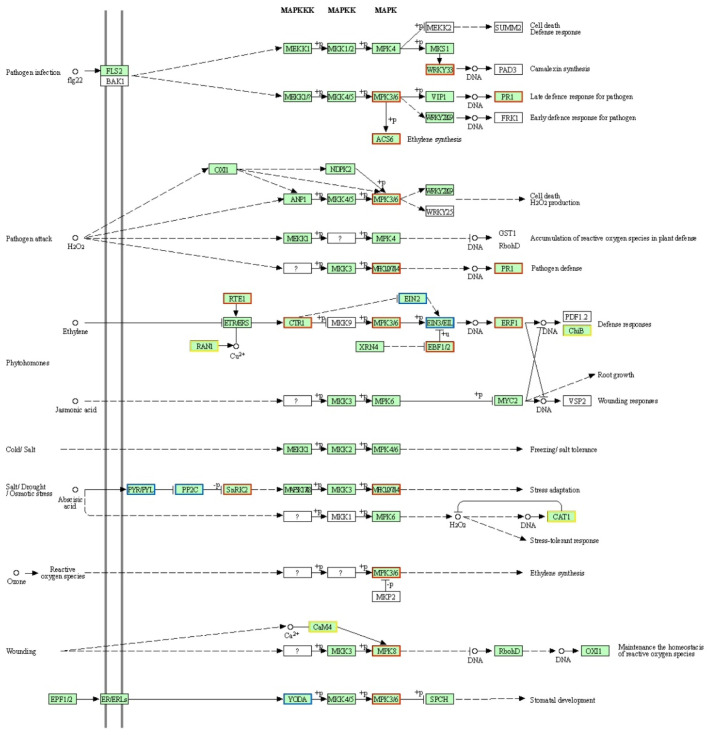
Alterations in the MAPK signaling pathway of FH2165 upon *M. oryzae* infection. Red boxes contain only upregulated genes; blue boxes contain only downregulated genes; yellow boxes contain both upregulated and downregulated genes. Image was downloaded from the KEGG PATHWAY Database (https://www.kegg.jp/kegg/pathway.html).

At 24 h after inoculation with *M. oryzae*, a substantial number of genes encoding MAP kinases, WRKY33 transcription factors, EBF1/2 (EIN3-binding F-box proteins), PR1 (pathogenesis-related protein 1), and cysteine proteases were upregulated in FH2165 (Figure 5). Conversely, a limited subset of genes, including those encoding EIN2/3 (ethylene-insensitive proteins) and BetvI allergen family proteins, was downregulated. Compared with its parents, FH2165 exhibited minimal differences in the upregulated genes. Notably, three genes (*Os05g0537400*, *Os01g0810300*, and *Os01g0846300*) were uniquely shared between FH2165 and HHSM. These genes predominantly regulate the expression of protein phosphatases and calmodulins, thereby modulating MAPK signal transduction. This observation suggested that FH2165 and HHSM could possess a specialized response mechanism mediated by these genes within the MAPK pathway, which potentially contributes to their enhanced resistance to blast disease.

### 2.7. Analysis of Plant Hormone Signal Transduction Pathway

**Figure 6:**
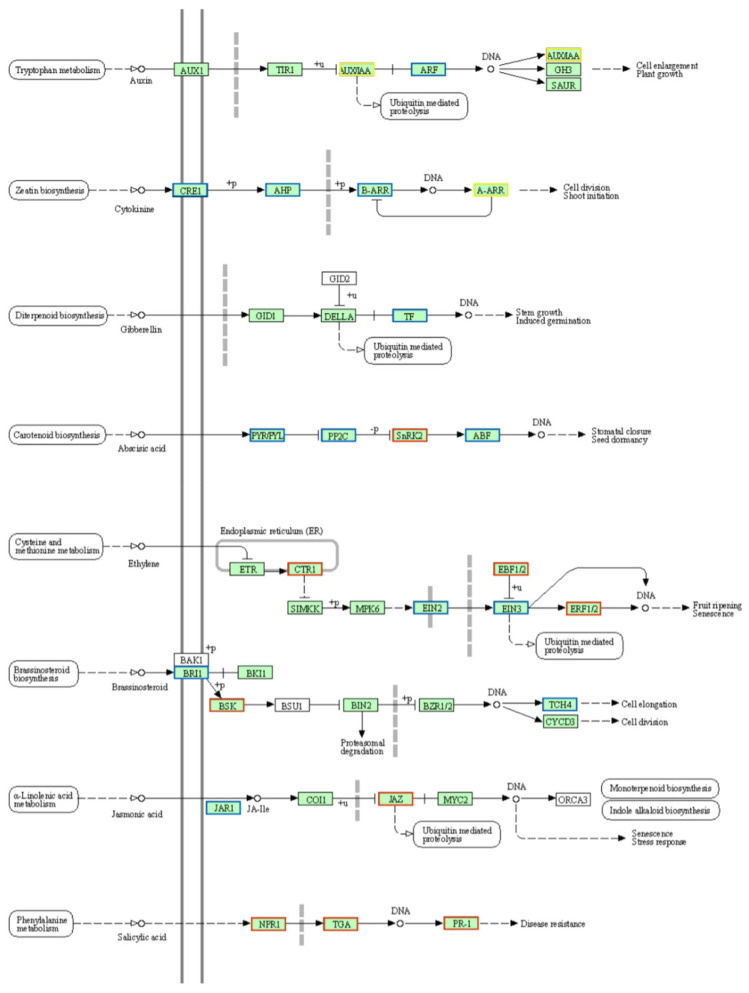
Alterations in the plant hormone signal transduction pathway of FH2165 upon *M. oryzae* infection. Red boxes contain only upregulated genes; blue boxes contain only downregulated genes; yellow boxes contain both upregulated and downregulated genes. Image was downloaded from the KEGG PATHWAY Database (https://www.kegg.jp/kegg/pathway.html).

The transduction of plant hormone signals is pivotal in mediating plant responses to various stresses and functions as a fundamental mechanism for regulating gene expression to bolster disease resistance [17]. Within the plant hormone signal transduction pathway, 49 DEGs were identified in FH2165, comprising 19 upregulated and 30 downregulated genes. HHSM exhibited 31 DEGs, with 13 upregulated and 18 downregulated, while MH86 had 39 DEGs, including 13 upregulated and 26 downregulated. Notably, SH527 displayed the highest number of enriched DEGs, totaling 56, with 19 upregulated and 37 downregulated. Across all four rice varieties examined, the prevalence of downregulated genes within this pathway was notably greater than that of upregulated genes, suggesting that the downregulated genes could exert a more substantial influence on rice blast resistance within the plant hormone signaling network. An analysis of FH2165 at 24 hpi revealed distinct expression patterns within the hormone signaling pathway: many genes encoding auxin-responsive proteins (Aux/IAA), which were involved in IAA transport, were downregulated (Figure 6). This downregulation subsequently affected the expression of auxin response factor (ARF) genes and the transcription of IAA-responsive genes.

Furthermore, genes encoding two-component response regulators ARR, Bet v I allergen family proteins, protein phosphatase 2C family proteins, eukaryotic transcription factors, EIN2/3, and GH3 auxin-responsive promoter family proteins exhibited varying degrees of downregulation (Figure 6). In contrast, the few upregulated genes were predominantly involved in the regulation of serine/threonine protein kinases, NPR1, and the transcription factor TGA6 (Figure 6). These findings suggested that rice plants can activate specific gene expression and promote protein synthesis through hormone-mediated signal transduction, thereby regulating physiological and biochemical processes such as cellular osmotic balance and antioxidant systems to enhance resistance against pathogens.

### 2.8. Analysis of the Unique DEGs in FH2165 and HHSM

Given that FH2165 and HHSM demonstrated superior blast resistance compared to MH86 and SH527, we hypothesized that these two rice varieties could possess specific genes that contribute to enhanced disease resistance. To investigate this hypothesis, we analyzed the DEGs unique to FH2165 and HHSM. Initially, we identified DEGs that were common to both FH2165 and HHSM, subsequently excluding those that were also expressed in MH86 and SH527. This screening process resulted in the identification of 86 unique co-expressed DEGs, comprising 43 upregulated and 43 downregulated genes (Table S14).

Then, further GO enrichment analysis of these 86 DEGs revealed three significant GO terms: RNA processing (GO:0006396), GTP binding (GO:0005525), and L-ascorbic acid binding (GO:0031418). Notably, RNA processing could indirectly facilitate rapid transcriptional reprogramming during *M. oryzae* infection by modulating gene expression and splicing patterns, thereby influencing the defense responses of plants [18,19]. Meanwhile, GTP binding is integral to the signal transduction functions of small GTPases and other GTP-binding proteins, which are pivotal in the regulation of cellular signaling networks and immune responses [20]. These proteins facilitate the perception and transmission of pathogen invasion signals in plants. Furthermore, L-ascorbic acid binding pertains to the molecular interactions with vitamin C, a crucial antioxidant in plants that neutralizes reactive oxygen species (ROS) generated during pathogen invasion, thereby maintaining intracellular redox homeostasis and modulating defense signal transduction [21]. Given that ROS levels escalate in rice cells following infection by *M. oryzae*, the precise regulation of ROS levels is essential for the activation of appropriate immune responses and the prevention of cellular damage [22]. Consequently, proteins exhibiting L-ascorbic acid binding activity could bolster disease resistance by regulating vitamin C utilization to modulate defense responses. Collectively, these three functional categories were likely implicated in rice blast resistance, particularly through their roles in mediating signal transduction and antioxidant defense.

Additionally, KEGG pathway analysis of the 86 co-expressed DEGs identified only three DEGs enriched in the purine metabolism pathway. Among these genes, *Os09g0442600* and *Os12g0515600* were upregulated in FH2165 and HHSM, whereas *Os11g0125900* was downregulated. Purine metabolism in rice plays a crucial role in energy supply, nucleic acid synthesis, and signal transduction [23]. Post-infection alterations in the expression of genes related to purine metabolism could indicate adaptive modifications to satisfy the energy requirements for defense responses and to provide precursors for the synthesis of defense molecules [24,25]. These findings highlight the potential roles of genotype-specific DEGs in RNA processing, GTP-mediated signaling, antioxidant regulation, and purine metabolism in enhancing blast resistance, laying the groundwork for future functional validation of key candidate genes to elucidate their precise mechanisms in FH2165 and HHSM resistance.

## 3. Discussion

FH2165 is an elite restorer line with superior agronomic traits (e.g., optimal plant architecture, high grain quality) and stable blast resistance, making it pivotal for hybrid rice breeding [9]. However, the molecular regulatory mechanisms underlying its resistance (especially how resistance-related traits are inherited and optimized from its parental lines) remain uncharacterized. This gap is notable because most prior transcriptomic studies on rice blast resistance have focused on either single varieties or known resistance gene-mediated pathways (e.g., NLR or RLK signaling) [10,11,12], with limited attention to the genetic basis of resistance in elite breeding lines and their parental inheritance patterns of resistance in these lines. Our transcriptomic analysis of FH2165 and its three parental lines (HHSM, MH86, SH527) at 0 and 24 h post-inoculation (hpi) addresses this gap by dissecting both conserved and genotype-specific resistance mechanisms, thereby deepening our understanding of rice blast resistance mechanisms.

A key conserved finding across all four genotypes was the enrichment of DEGs in carbon metabolism, amino acid biosynthesis, and redox pathways—consistent with Devanna et al. (2022) and Jain et al. (2019), who reported that energy metabolism reprogramming and secondary metabolite synthesis are core defensive responses to *M. oryzae* [14,26]. However, our study advances this knowledge by revealing genotype-specific optimization of this conserved strategy: FH2165 and HHSM (the two more resistant varieties) showed more targeted upregulation of DEGs in the pentose phosphate pathway (a source of NADPH for ROS scavenging) and phenylalanine biosynthesis (a precursor for lignin, which reinforces cell walls) compared to the susceptible MH86 and SH527. This suggested that resistance in FH2165 is not merely a general activation of metabolic pathways, but a refined tuning of energy allocation toward more cost-effective defensive processes.

Another critical insight is the unique ribosome pathway response that distinguishes HHSM from FH2165, MH86, and SH527. While FH2165, MH86, and SH527 exhibited significant upregulation of ribosome-related DEGs (likely to enhance the synthesis of defense proteins such as SPR9 [27]), HHSM showed few DEGs in this pathway and maintained ribosomal stability. This contrasts with He et al. (2023), who linked ribosomal pathway activation to broad-spectrum resistance in rice [27]. Our findings suggest an alternative resistance strategy in HHSM: rather than relying on de novo protein synthesis, it could utilize pre-stored defensive proteins or structural barriers (e.g., thicker cell walls). Importantly, FH2165, which inherits resistance from HHSM, did not adopt HHSM’s ribosomal stability strategy but instead combined HHSM’s metabolic refinement with the ribosome-mediated protein synthesis of MH86 and SH527. Such a hybrid regulatory pattern explains FH2165’s balanced performance (high resistance coupled with superior agronomic traits) and provides a molecular basis for understanding resistance inheritance in breeding lines.

The analysis of 86 unique co-expressed DEGs in FH2165 and HHSM further extends current knowledge. GO enrichment analysis identified three key functions: GTP binding, L-ascorbic acid binding, and RNA processing. While GTP-binding proteins (e.g., Rab GTPases) have been implicated in NLR protein trafficking [20], our study is the first to link their specific upregulation in an elite restorer line to enhanced resistance against *M. oryzae*. Similarly, L-ascorbic acid binding proteins—known to regulate ROS homeostasis [21]—were found to be co-expressed with GTP-binding proteins in FH2165 and HHSM, suggesting a coordinated module for ROS scavenging and signal transduction. This is distinct from Kou et al. (2019), who only reported ROS bursts in susceptible varieties [22]; our work reveals that resistant varieties actively fine-tune ROS levels Via L-ascorbic acid metabolism, rather than merely suppressing such bursts. For RNA processing, prior studies have focused on its role in general stress responses [19], but our data suggest it may specifically regulate the splicing of resistance genes (e.g., MAPK pathway components) in FH2165—supported by the co-expression of RNA processing-related DEGs with MAPK-related upregulated genes.

KEGG analysis of these unique DEGs also uncovered a previously unrecognized role of purine metabolism in FH2165’s resistance. While Sun et al. (2024) reported the involvement of purine metabolism in fungal pathogenicity [23], our study is the first to establish a link between rice’s purine metabolism (Via DEGs such as *Os09g0442600*) and resistance against *M. oryzae*, specifically in rice restorer lines. The upregulation of purine metabolism genes in FH2165 and HHSM likely supports not only energy supply (Via ATP) but also the synthesis of defensive nucleotides (e.g., cyclic AMP for signal transduction), thereby enriching our understanding of how primary metabolism contributes to resistance in breeding lines.

One limitation of our study is the absence of functional validation for key candidate genes (e.g., GTP-binding or purine metabolism-related genes), which will be addressed in future work Via gene knockout or overexpression assays. Additionally, while we focused on 24 hpi (a critical infection stage [14,15]), extending the sampling to longer time points could reveal late-stage resistance mechanisms (e.g., the maintenance of hypersensitive response).

## 4. Materials and Methods

### 4.1. Plant Materials, Growth Conditions, and Treatments

The rice varieties FH2165, HHSM, MH86, and SH527 were maintained by Rice Research Institute, Fujian Academy of Agricultural Sciences, China. The seeds underwent surface sterilization, soaking, and germination prior to cultivation in a greenhouse under controlled environmental conditions (25–28 °C, 16 h light/8 h dark, and relative humidity exceeding 80%) until they reached the three-leaf and one-heart developmental stage. *M. oryzae* strains were isolated and purified from diseased samples collected in the field. Mycelial subcultures were grown on the Complete Medium (CM: 10 g/L sucrose, 6 g/L yeast extract, 6 g/L casamino acids, 20 g/L agar), while spore induction was conducted on Rice Bran Agar Medium (RBA: 20 g/L rice bran, 20 g/L sucrose, 20 g/L agar, pH 6.0–6.5) [28,29]. Spores were harvested by rinsing RBA cultures with a sterile 0.02% Tween 20 solution, filtered through sterile filter paper, and adjusted to a concentration of 0.5–1.0 × 10^5^ spores/mL using a hemocytometer [30]. The resulting spore suspension was uniformly applied to the leaves of the rice seedlings via spraying. Inoculated plants were incubated in a dark, moist chamber at 25 °C for 24 h, then transferred to a greenhouse for further cultivation. Leaf tissues were collected from each cultivar at 0 h and 24 h post-inoculation (hpi). To ensure data reliability, three biological replicates were established for each genotype. All replicates were inoculated with the same batch of spore suspension to ensure treatment consistency. Phenotypic responses, including lesion development, were photographed and recorded before and after inoculation.

### 4.2. RNA Isolation and Library Preparation

Leaf samples were obtained from four rice varieties at two time points: 0 hpi (prior to *M. oryzae* inoculation, designated as FHMO_0, HHMO_0, MHMO_0, SHMO_0) and 24 hpi (designated as FHMO_24, HHMO_24, MHMO_24, SHMO_24). For each sample, leaves from five plants were harvested and pooled into a composite sample to minimize the effect of transcriptome unevenness among plants and ensure biological representativeness. For each sample, leaves exhibiting uniform growth were selected for total RNA extraction, and total RNA was extracted using the TRIzol reagent method (Invitrogen, Carlsbad, CA, USA) [31]. The quality of the extracted RNA was assessed through a tripartite evaluation process: integrity was verified via agarose gel electrophoresis; purity was measured using NanoDrop spectrophotometry (Nanodrop Technologies, Inc., Wilmington, DE, USA); and fragment size distribution was analyzed with the Agilent 2100 Bioanalyzer (Agilent Technologies, Inc., Santa Clara, CA, USA) [32]. Subsequently, mRNA was isolated using Oligo(dT)-conjugated magnetic beads and fragmented into shorter segments using a fragmentation buffer (NEB, Ipswich, MA, USA). First-strand cDNA was synthesized through reverse transcription employing random hexamer primers, followed by the synthesis of second-strand cDNA using DNA polymerase I, buffer, and deoxynucleotide triphosphates (dNTPs). The double-stranded cDNA underwent purification utilizing AMPure XP beads (Beckman Coulter, Beverly, MA, USA), followed by end repair, adenylation at the 3′ ends (A-tailing), and adapter ligation. Subsequently, fragment size selection was also performed using AMPure XP beads. The selected fragments were then amplified through PCR to construct cDNA libraries. The quality of these libraries was assessed by verifying the fragment size with an Agilent 2100 Bioanalyzer and accurately quantifying the effective concentration, ensuring it exceeded 4 nM, using qPCR. Paired-end transcriptome sequencing was executed on an Illumina NovaSeq 6000 platform (Illumina, Hayward, CA, USA), employing reference-based analysis. RNA isolation, library preparation, and sequencing were performed by the Beijing Allwegene Technology Company Limited (Beijing, China).

### 4.3. Sequencing Quality Control and Read Alignment

Raw sequencing data frequently contain adapter contaminants and low-quality reads, which can interfere with subsequent analyses. To address this, quality control filtering was conducted using Trimmomatic software (version 0.33) based on the following criteria: (1) removal of reads containing adapter sequences; (2) exclusion of reads with more than 10% ambiguous bases (N); and (3) elimination of reads with more than 50% of bases having a Phred quality score (Q) of 20 or lower [33]. The resulting high-quality reads were then aligned to the rice reference genome (*Oryza sativa Japonica* Group IRGSP-1.0; accessible at http://rice.plantbiology.msu.edu/, accessed on 13 October 2025) utilizing the STAR (Spliced Transcripts Alignment to a Reference) software (version 2.6) [34]. 

### 4.4. Differentially Expressed Genes Screening and Functional Annotation

Gene expression levels were normalized using Fragments Per Kilobase of transcript per Million mapped reads (FPKM) to facilitate comparisons across genes of different lengths and samples with different sequencing depths. Genes with an FPKM value of ≥1 were classified as expressed [35]. Differentially expressed genes (DEGs) were identified through pairwise comparisons between *M. oryzae*-inoculated samples at 24 h post-inoculation (MO_24, treatment) and non-inoculated controls at 0 h (MO_0) for each variety, utilizing the DEGseq software (version 1.62.0). To minimize experimental errors and ensure the reliability of identified DEGs, strict quality control was applied during data analysis. Specifically, DEGs were screened using the following criteria: |log_2_(Fold Change)| > 1 and q-value (padj) < 0.005 [36]. Functional annotation of DEGs was conducted using the Gene Ontology (GO) and Kyoto Encyclopedia of Genes and Genomes (KEGG) databases. GO enrichment analysis was performed using GOseq software (version 1.36.0), with significant enrichment determined by a *p*-value ≤ 0.05 [37]. For KEGG pathway analysis, significantly enriched pathways were ranked by *Q*-value, with *Q* ≤ 0.05 considered statistically significant; lower *Q*-values indicate a higher degree of enrichment significance [38].

### 4.5. Validation of RNA-Seq by qRT-PCR

To validate the RNA-Seq results, quantitative real-time PCR (qRT-PCR) was performed on 10 randomly selected DEGs. The experiments were performed using a Chromo 4 real-time PCR detection system (Bio-Rad, Hercules, CA, USA). Diluted cDNA templates were amplified with gene-specific primers using SYBR Green real-time PCR master mix (Toyobo, Osaka, Japan). The rice *Actin1* gene served as the internal reference for normalization [39]. The sequences of primers used for qRT-PCR are listed in Table S1.

## 5. Conclusions

This study systematically elucidated the blast resistance mechanisms in the elite rice restorer line FH2165 and its parental lines. Transcriptome analysis revealed relatively conserved expression patterns following *M. oryzae* infection, with DEGs predominantly enriched in carbon metabolism, amino acid biosynthesis, and redox pathways, reflecting a defense strategy based on energy reprogramming and secondary metabolite synthesis. The unique co-expressed DEGs shared by FH2165 and HHSM highlighted GTP-binding-mediated NLR receptor trafficking and L-ascorbate-related antioxidant defense mechanisms. These findings clarified the resistance basis of FH2165 and provided candidate targets for breeding high-yield, disease-resistant hybrid rice.

## Figures and Tables

**Table 1:** Summary of Illumina transcriptome reads aligned to the reference genome.

Sample	Raw Reads	Clean Reads	Q20	Q30	GC Content	Uniquely Mapped
FHMO_0	51,407,562	47,124,988	98.21%	94.65%	49.35%	43,535,238 (92.38%)
FHMO_24	50,904,432	47,060,074	98.18%	94.56%	48.15%	43,296,564 (92.00%)
HHMO_0	49,472,538	44,541,728	98.20%	94.67%	49.11%	40,984,592 (92.01%)
HHMO_24	51,106,482	46,917,842	98.15%	94.51%	48.26%	42,758,974 (91.14%)
MHMO_0	48,331,684	44,533,256	98.25%	94.78%	49.20%	41,411,002 (92.99%)
MHMO_24	49,630,466	45,871,454	98.24%	94.76%	48.25%	42,282,068 (92.18%)
SHMO_0	46,636,886	41,370,362	98.27%	94.83%	49.66%	38,016,724 (91.89%)
SHMO_24	47,496,244	43,262,282	98.24%	94.71%	48.57%	39,659,960 (91.67%)

## Data Availability

The original contributions presented in this study are included in the article/Supplementary Material. Further inquiries can be directed to the corresponding authors.

## Supplementary Materials

The following supporting information can be downloaded at: https://www.mdpi.com/article/10.3390/ijms262010164/s1.
