# Supplementary figures and images for: Comprehensive Transcriptomic Analysis of the Molecular Mechanisms Conferring Resistance to Rice Blast in the Elite Restorer Line Fuhui2165
Source: Int J Mol Sci. 2025 Oct 19;26(20):10164. doi: 10.3390/ijms262010164 (PMC12564515; doi:10.3390/ijms262010164)

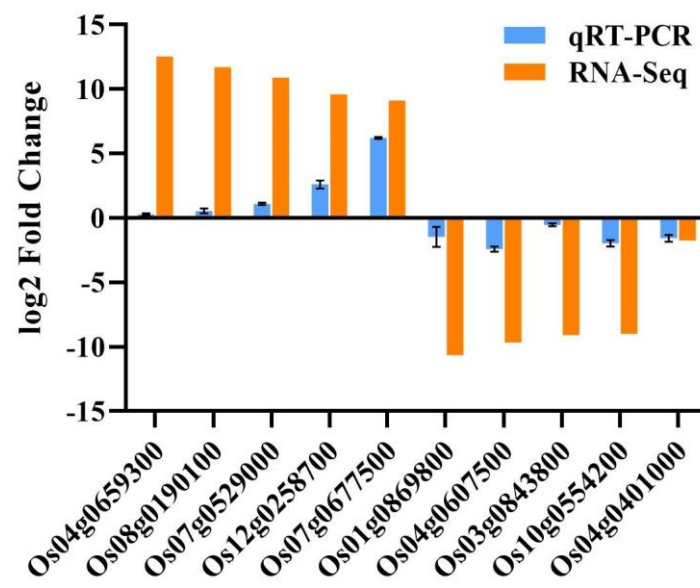

**Figure S1.** Validation of RNA-Seq results by qRT-PCR.

Supplement: Supplementary file 1 [file ijms-26-10164-s001.zip › Figure S1.pdf]
